# Supplementary material for: Cortical idiosyncrasies predict the perception of object size
Source: Nat Commun. 2016 Jun 30;7:12110. doi: 10.1038/ncomms12110 (PMC4931347; doi:10.1038/ncomms12110)
Supplement: Supplementary Information — Supplementary Figures 1 - 4, Supplementary Tables 1 and 2 and Supplementary Notes 1 - 3 [file ncomms12110-s1.pdf]

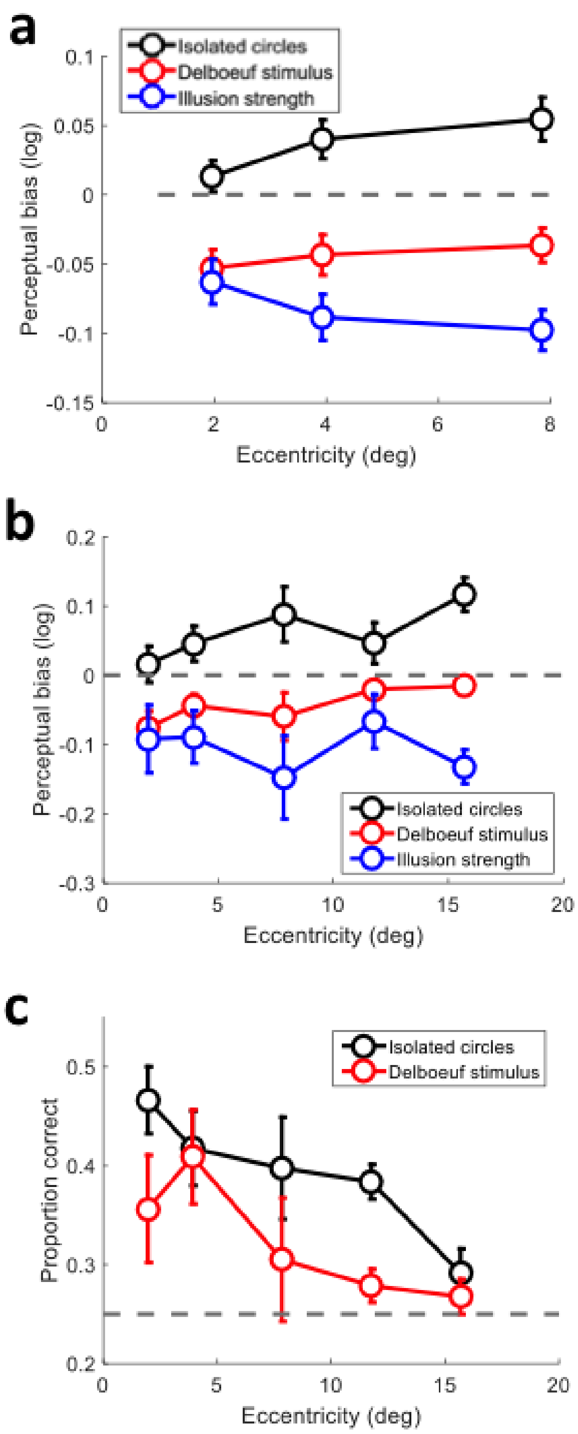

Supplementary Figure 1. a. Average perceptual bias (positive and negative: target appears smaller or larger than reference, respectively) weighted by the acuity (reciprocal of squared dispersion), across individuals plotted against target eccentricity for simple isolated circles (black), contextual Delboeuf stimuli (red), and relative illusion strength (blue), that is, the difference in biases measured for the two stimulus conditions. Data from all 10 observers in the size eccentricity bias experiment. **b.** Data from 4 observers in the size far-eccentricity bias experiment. **c.** Behavioral accuracy on the task for the 4 observers in the size far-eccentricity bias experiment. Chance was 25% and is noted by the dashed grey line. In all plots, error bars denote ±1 standard error of the mean.

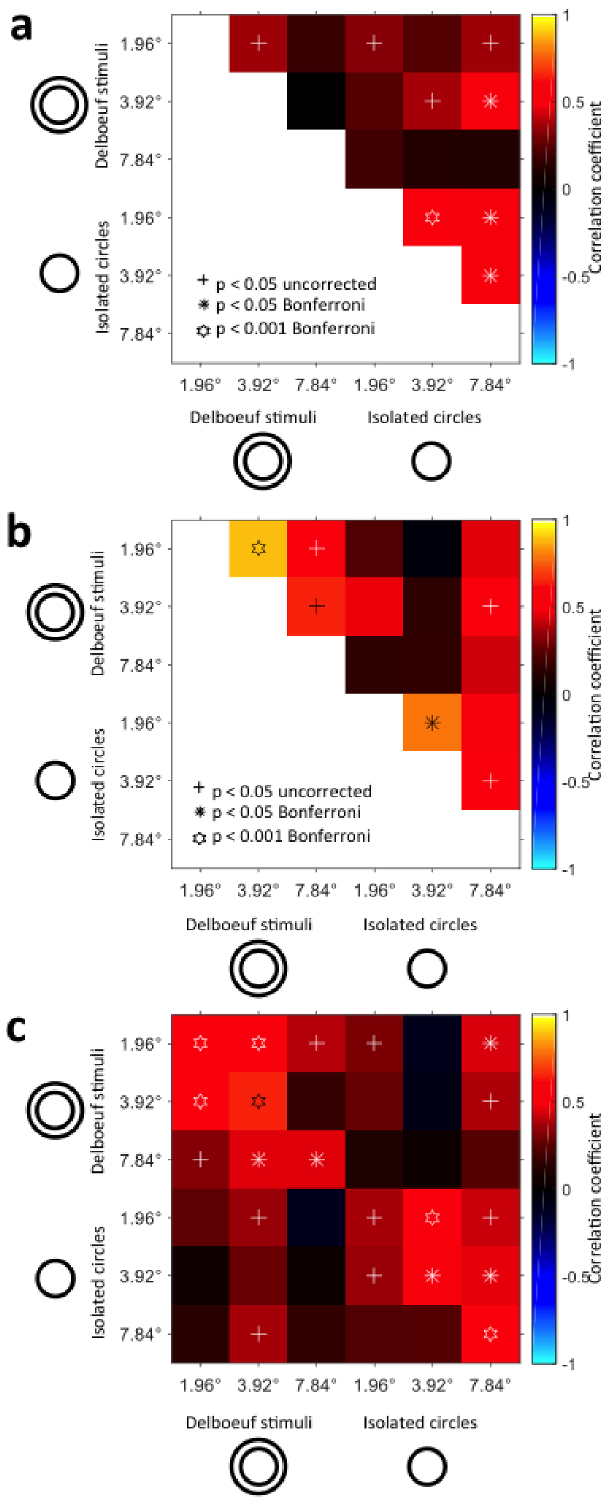

Supplementary Figure 2. Correlation matrices showing the relationship between the perceptual biases in the two conditions (isolated circles and Delboeuf stimuli) and at the three stimulus eccentricities. **a.** Correlations after removing between-subject variance, i.e. the mean across the biases for the four targets was subtracted from each condition. **b.** Correlations after removing the within-subject variance, i.e. biases were averaged across the four targets in each condition. **c.** Correlations between the first and second session of the experiment conducted on different days. All other conventions as in Figure 1e. Note that statistical power in **b** is lower relative to the other figures, because after averaging there is only a quarter of the number of observations.

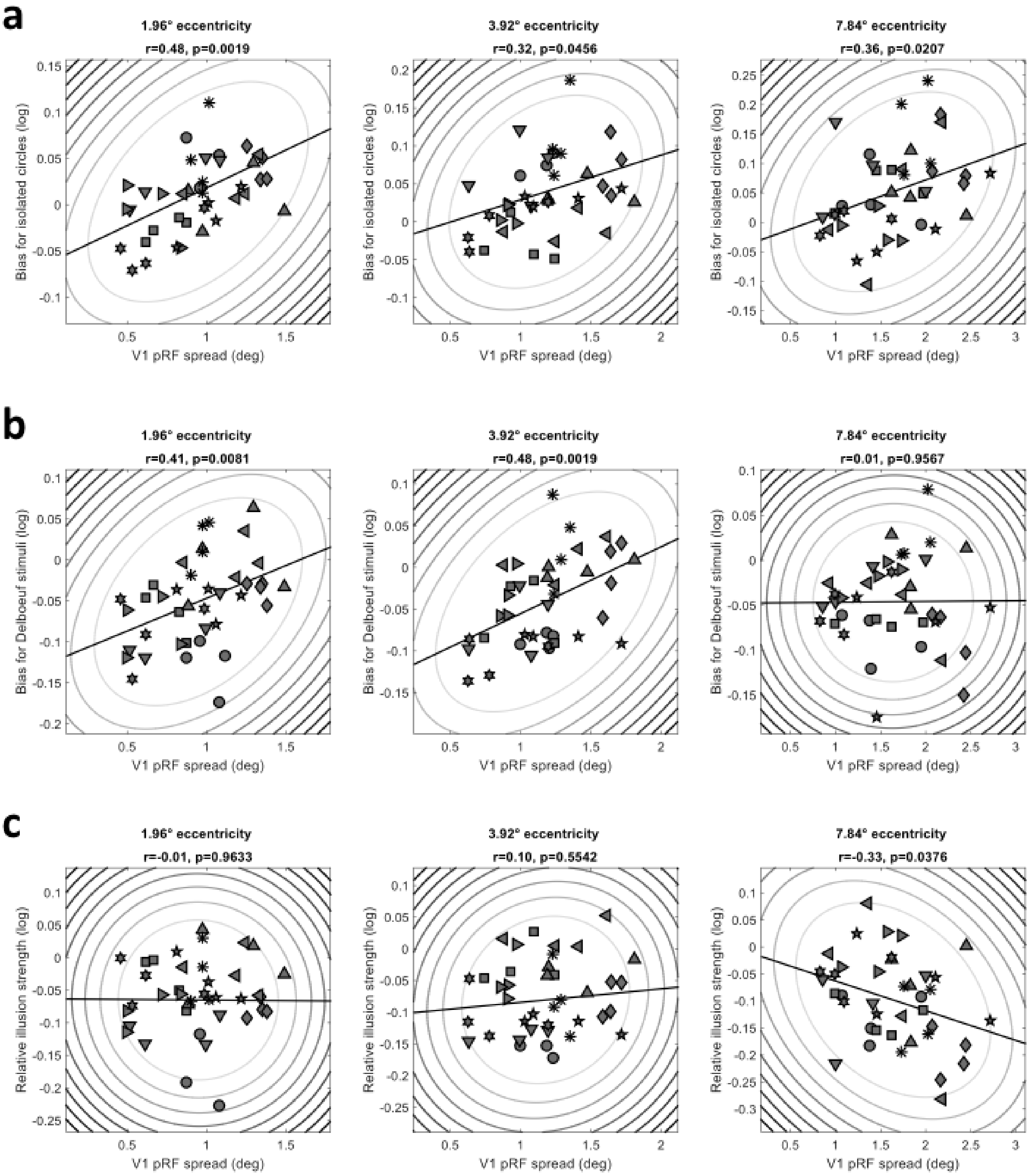

Supplementary Figure 3. Perceptual biases for isolated circles (**a**), for the Delboeuf stimuli (**b**), and the relative illusion strength (**c**), that is, the bias for Delboeuf stimuli minus the bias for isolated circles, plotted against pRF spread at the corresponding location in V1 for each observer and stimulus location. Columns show data for stimuli at 1.96°, 3.92°, or 7.84° eccentricity. Symbols denote individual observers. Elliptic contours denote the Mahalanobis distance from the bivariate mean. The straight, black lines denote the linear regression.

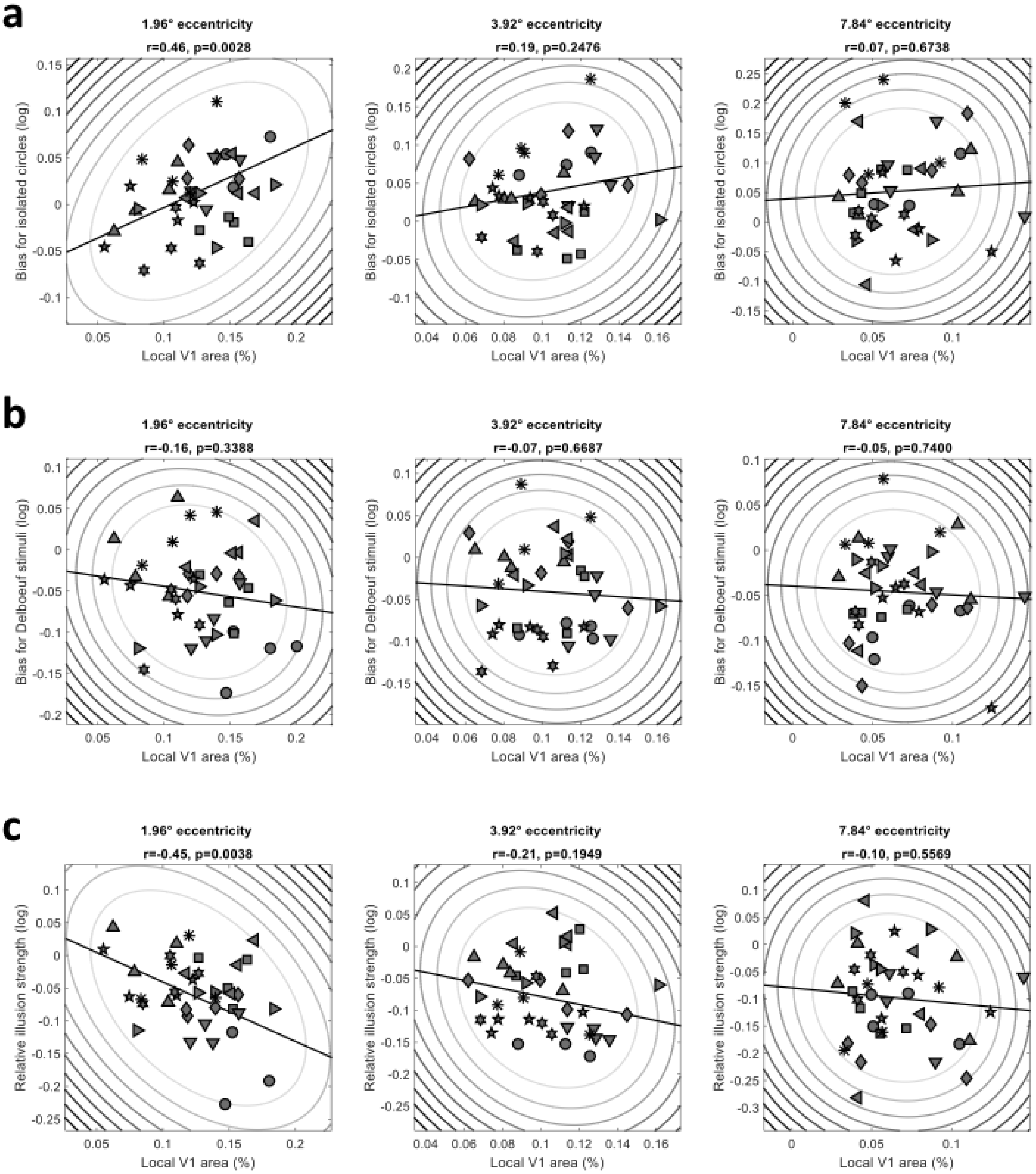

Supplementary Figure 4. Perceptual biases for isolated circles (**a**), for the Delboeuf stimuli (**b**), and the relative illusion strength (**c**), that is, the bias for Delboeuf stimuli minus the bias for isolated circles, plotted against the surface area of the corresponding location in V1 for each observer and stimulus location (as percentage of the area of the whole cortical hemisphere). Columns show data for stimuli at 1.96°, 3.92°, or 7.84° eccentricity. Symbols denote individual observers. Elliptic contours denote the Mahalanobis distance from the bivariate mean. The straight, black lines denote the linear regression.

**Pooled eccentricities**	**V1 pRF spread vs**		
	Isolated circles	*Delboeuf stimuli*	*Illusion index*
Pooled data (main analysis)	R = 0.43, p < 0.001, n = 120	R = 0.21, p = 0.022, n = 120	R = -0.22, p = 0.017, n = 120
Within-subject variance only	R = 0.29, p = 0.001, n = 120	R = 0.15, p = 0.112, n = 120	R = -0.16, p = 0.077, n = 120
Second-level analysis	R = 0.41, t(29) = 2.81, p = 0.009	R = 0.25, t(29) = 1.54, p = 0.135	R = -0.06, t(29) = -0.37, p = 0.714
**1.96° eccentricity**	**V1 pRF spread vs**		
	Isolated circles	*Delboeuf stimuli*	*Illusion index*
Pooled data (main analysis)	R = 0.48, p = 0.002, n = 40	R = 0.41, p = 0.008, n = 40	R = -0.01, p = 0.963, n = 40
Within-subject variance only	R = 0.37, p = 0.018, n = 40	R = 0.19, p = 0.231, n = 40	R = -0.16, p = 0.325, n = 40
Second-level analysis	R = 0.57, t(9) = 1.96, p = 0.082	R = 0.10, t(9) = 0.35, p = 0.735	R = -0.27, t(9) = -0.90, p = 0.391
**3.92° eccentricity**	**V1 pRF spread vs**		
	Isolated circles	*Delboeuf stimuli*	*Illusion index*
Pooled data (main analysis)	R = 0.32, p = 0.046, n = 40	R = 0.48, p = 0.002, n = 40	R = 0.10, p = 0.554, n = 40
Within-subject variance only	R = 0.21, p = 0.196, n = 40	R = 0.23, p = 0.146, n = 40	R = -0.02, p = 0.917, n = 40
Second-level analysis	R = 0.41, t(9) = 2.30, p = 0.047	R = 0.40, t(9) = 1.52, p = 0.163	R = 0.11, t(9) = 0.39, p = 0.705
**7.84° eccentricity**	**V1 pRF spread vs**		
	Isolated circles	*Delboeuf stimuli*	*Illusion index*
Pooled data (main analysis)	R = 0.36, p = 0.021, n = 40	R = 0.01, p = 0.957, n = 40	R = -0.33, p = 0.038, n = 40
Within-subject variance only	R = 0.29, p = 0.066, n = 40	R = 0.10, p = 0.546, n = 40	R = -0.21, p = 0.203, n = 40
Second-level analysis	R = 0.21, t(9) = 0.78, p = 0.456	R = 0.24, t(9) = 0.76, p = 0.467	R = 0.00, t(9) = 0.01, p = 0.988

Supplementary Table 1. All correlations between pRF spread in V1 and perceptual bias measures using four complementary analysis approaches: 'Pooled data' refers to the main analysis presented in which we simply treated each of the 12 visual field locations per observer as an separate data point. 'Within-subject variance only' refers to the equivalent analysis after removing the mean pRF spread and perceptual bias, respectively, across the four locations for each eccentricity and observer. 'Second-level analysis' refers to the analysis in which we first calculated the correlation across four locations separately for each observer and eccentricity and then determined whether the average correlation (after z-transformation) was different from zero. Both, the average correlation coefficient and the statistics of the t-test against zero are shown. Only the full combination of all four visual field locations per observer and eccentricity are used. Across the table, cells shaded in grey denote correlations statistically significant at p<0.05.

**Pooled eccentricities**	**V1 surface area vs**		
	Isolated circles	*Delboeuf stimuli*	*Illusion index*
Pooled data (main analysis)	R = -0.00, p = 0.965, n = 120	R = -0.09, p = 0.315, n = 120	R = -0.06, p = 0.507, n = 120
Within-subject variance only	R = 0.21, p = 0.022, n = 120	R = 0.13, p = 0.167, n = 120	R = -0.10, p = 0.254, n = 120
Second-level analysis	R = 0.44, t(29) = 2.63, p = 0.014	R = 0.31, t(29) = 1.97, p = 0.059	R = -0.11, t(29) = -0.73, p = 0.470
**1.96° eccentricity**	**V1 surface area vs**		
	Isolated circles	*Delboeuf stimuli*	*Illusion index*
Pooled data (main analysis)	R = 0.46, p = 0.003, n = 40	R = -0.16, p = 0.339, n = 40	R = -0.45, p = 0.004, n = 40
Within-subject variance only	R = 0.43, p = 0.005, n = 40	R = 0.38, p = 0.017, n = 40	R = -0.06, p = 0.708, n = 40
Second-level analysis	R = 0.47, t(9) = 2.22, p = 0.054	R = 0.59, t(9) = 2.44, p = 0.037	R = 0.21, t(9) = 0.89, p = 0.395
**3.92° eccentricity**	**V1 surface area vs**		
	Isolated circles	*Delboeuf stimuli*	*Illusion index*
Pooled data (main analysis)	R = 0.19, p = 0.248, n = 40	R = -0.07, p = 0.669, n = 40	R = -0.21, p = 0.195, n = 40
Within-subject variance only	R = 0.25, p = 0.116, n = 40	R = 0.03, p = 0.847, n = 40	R = -0.22, p = 0.177, n = 40
Second-level analysis	R = 0.47, t(9) = 1.19, p = 0.265	R = 0.17, t(9) = 0.84, p = 0.423	R = -0.39, t(9) = -1.25, p = 0.244
**7.84° eccentricity**	**V1 surface area vs**		
	Isolated circles	*Delboeuf stimuli*	*Illusion index*
Pooled data (main analysis)	R = 0.07, p = 0.674, n = 40	R = -0.05, p = 0.740, n = 40	R = -0.10, p = 0.557, n = 40
Within-subject variance only	R = 0.09, p = 0.584, n = 40	R = -0.01, p = 0.957, n = 40	R = -0.08, p = 0.618, n = 40
Second-level analysis	R = 0.37, t(9) = 1.46, p = 0.178	R = 0.10, t(9) = 0.30, p = 0.775	R = -0.13, t(9) = -0.76, p = 0.466

Supplementary Table 2. All correlations between cortical surface area in V1 and perceptual bias measures using four complementary analysis approaches: 'Pooled data' refers to the main analysis presented in which we simply treated each of the 12 visual field locations per observer as an separate data point. 'Within-subject variance only' refers to the equivalent analysis after removing the mean cortical surface area and perceptual bias, respectively, across the four locations for each eccentricity and observer. 'Second-level analysis' refers to the analysis in which we first calculated the correlation across four locations separately for each observer and eccentricity and then determined whether the average correlation (after z-transformation) was different from zero. Both, the average correlation coefficient and the statistics of the t-test against zero are shown. Only the full combination of all four visual field locations per observer and eccentricity are used. Across the table, cells shaded in grey denote correlations statistically significant at $p<0.05$.

Supplementary Note 1

Reliability of perceptual bias estimates

We further confirmed the reliability of these bias estimates by comparing estimates from two sessions conducted on different days (Supplementary Figure 2c). Moreover, 9 of our observers were tested twice, with approximately one year between sessions. Despite the long time between experiments and variation in the stimulus sampling procedure (see Methods), estimates of perceptual biases at target eccentricity 3.92° (which was common to both experiments) were correlated (Pearson's $r=0.35$, $p=0.0373$, $n=36$). This correlation was largely driven by the within-subject variance, and was considerably greater after subtracting the mean across the four target locations for every condition ($r=0.58$, $p=0.0002$, $n=36$). In contrast, removing the within-subject variance by averaging bias estimates across the four targets reduced the correlation substantially ($r=0.18$, $p=0.6483$, $n=36$). Finally, 4 observers repeated the experiment two years after the initial experiment, allowing us to compare biases for the three eccentricities tested in the original experiment ($n=48$). We again found a strong reliability of idiosyncratic biases ($r=0.47$, $p=0.001$, $n=48$; after removing between-subject variance: $r=0.71$, $p<0.001$, $n=48$).

Supplementary Note 2

Intra-individual differences analysis

For each observer we obtained separate measures of perceptual bias and cortical measures corresponding to 12 visual field locations. We then calculated correlations by comparing all locations (120 data points) or across quadrants but separately for each eccentricity (40 data points). Naturally, multiple observations for a given participant are not strictly independent. Therefore, as described in the main text we performed three parallel analyses:

1. Pooled data (all variance): The main analyses reported in our study simply show the pooled data without any additional processing. They therefore compare the 120 (or 40, when separating eccentricities) data points with each visual field location as a separate data point (Figure 2 c-e; Figure 5). This approach is the most inclusive as it incorporates both the within-subject variance (the pattern of variability across visual field locations) as well as the conventional between-subject variance (differences between individual observers that affect all visual field locations in a given observer equally). Our hypothesis that cortical idiosyncrasies in pRF spread/surface area relate to perceptual biases suggests that both between- and within-subject variance should contribute similarly to the correlation.

2. Within-subject variance only: We also calculated correlations after removing the between-subject variance by first subtracting the mean of measurements across the four visual field locations from each eccentricity and observer. This way the correlation only takes into account the variability across quadrants within each observer/eccentricity.
3. Second-level analysis of within-subject variance: In an alternative analysis using only the within-subject variance we calculated the correlation between the two variables separately for each eccentricity and each observer, and then determined whether the average correlation (after Fisher's z-transformation to linearize r) is significantly different from zero using a one-sample t-test. However, this approach is comparably underpowered because it relies on only four data points (one per visual field quadrant) for each observer and eccentricity. Thus, each individual correlation coefficient is likely to be skewed by outliers or individual unreliable measurements and this approach is prone to both type I and type II error.

Supplementary Note 3

Power analysis

To confirm the validity of our analysis approach, we conducted simulations to determine its statistical power. In 10,000 simulations we generated random data sets with the same sample sizes and dimensionality of our data to test three situations: A) Complete null hypothesis: the two variables were completely uncorrelated. B) Complete alternative hypothesis: The 120 data points were chosen from the same underlying distribution with a population correlation of 0.3. This is the alternative hypothesis we seek to test in this study, because it assumes that variability in cortical measures (pRF spread or cortical surface area) is directly linked to perceptual biases. C) Between-subject relationship only: two variables of 10 subjects with 12 stimulus locations were correlated (using population correlation of 0.3) but the within-subject variance was random noise (Gaussian noise with 0.5 standard deviations) added to the 4 observations for each observer and eccentricity. This situation assumes the effect is solely driven by the between-subject variance and within-subject variance is merely measurement noise within each observer.

These simulations showed that analyses 1 and 3 (see Supplementary Note 2) have nominal levels of false positives (~5%) when the null hypothesis is true (situation A), but analysis 2 (within-subject variance only) somewhat inflates false positive rates to around 9%. Conversely, for situation B when the alternative hypothesis is true and there is a direct relationship between the two variables, analyses 1 and 2 are most sensitive with a statistical power of approximately 92% and 89%,

respectively. Analysis 3 was far less sensitive (65% power). Finally, in situation C when the relationship is only driven by the between-subject variance, statistical power for analysis 1 is still moderately high (65%) but as expected power for all the analyses is much lower (9% for analysis 2, respectively, and at the alpha level of 5% for analysis 3). Thus if our hypothesis of a direct link of the within-subject variability in perceptual biases and V1 measures were untrue and the relationship was mainly driven by between-subject variance, we would have been unlikely to detect any correlations in these control analyses. This is clearly not the case as the pattern of results is qualitatively very similar between the four analyses in most cases – especially the main result comparing pRF spread to perceptual biases of isolate circles is highly significant in all four analyses (Supplementary Tables 1 and 2).
