# Supplementary figures and images for: Cortical idiosyncrasies predict the perception of object size
Source: Nat Commun. 2016 Jun 30;7:12110. doi: 10.1038/ncomms12110 (PMC4931347; doi:10.1038/ncomms12110)

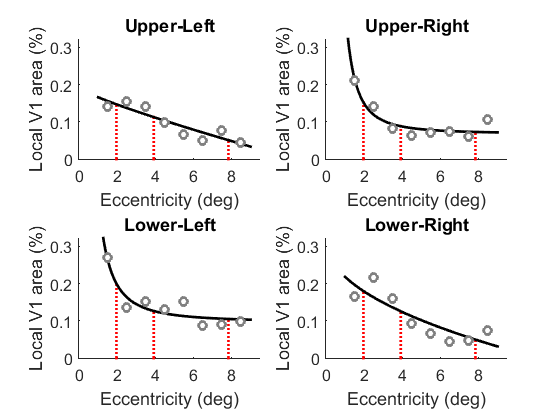

Supplement: Supplementary Data 1 — Each PNG file plots the mean V1 pRF spread (*-sigma.png) or the local surface area (*-area.png) in V1 for the four visual field quadrants against eccentricity bands that are 1° in width. The vertical dashed red lines indicate the eccentricities of the target stimuli in the psychophysical experiments. The solid black lines denote the fitted polynomial functions.The associated XLS files contain the mean pRF spread (*-sigma.xls) or local surface area (*-area.xls) in V1 plotted for each eccentricity band in these plots. There are four worksheets in each file, one for each visual field quadrant. The first column in each sheet is the eccentricity of each band. The second column is the mean pRF spread or local surface area of that eccentricity band. In the *-sigma.xls files the third and fourth columns are the length of the error bars, which correspond to the bootstrapped 95% confidence intervals. [file ncomms12110-s2.zip › Individuals/S1-area.png]

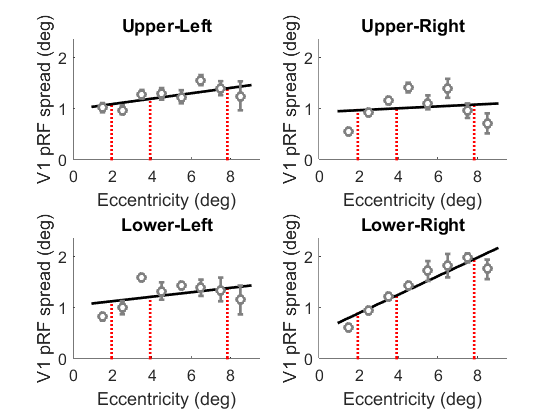

Supplement: Supplementary Data 1 — Each PNG file plots the mean V1 pRF spread (*-sigma.png) or the local surface area (*-area.png) in V1 for the four visual field quadrants against eccentricity bands that are 1° in width. The vertical dashed red lines indicate the eccentricities of the target stimuli in the psychophysical experiments. The solid black lines denote the fitted polynomial functions.The associated XLS files contain the mean pRF spread (*-sigma.xls) or local surface area (*-area.xls) in V1 plotted for each eccentricity band in these plots. There are four worksheets in each file, one for each visual field quadrant. The first column in each sheet is the eccentricity of each band. The second column is the mean pRF spread or local surface area of that eccentricity band. In the *-sigma.xls files the third and fourth columns are the length of the error bars, which correspond to the bootstrapped 95% confidence intervals. [file ncomms12110-s2.zip › Individuals/S1-sigma.png]

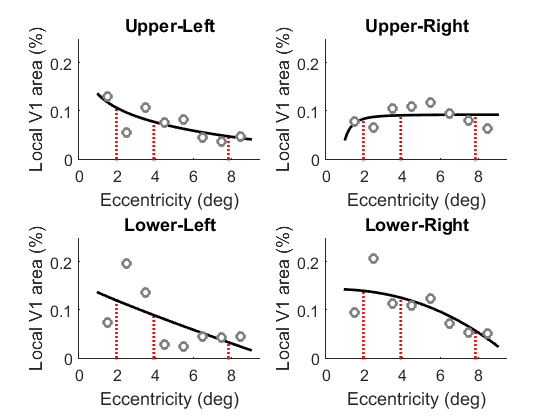

Supplement: Supplementary Data 1 — Each PNG file plots the mean V1 pRF spread (*-sigma.png) or the local surface area (*-area.png) in V1 for the four visual field quadrants against eccentricity bands that are 1° in width. The vertical dashed red lines indicate the eccentricities of the target stimuli in the psychophysical experiments. The solid black lines denote the fitted polynomial functions.The associated XLS files contain the mean pRF spread (*-sigma.xls) or local surface area (*-area.xls) in V1 plotted for each eccentricity band in these plots. There are four worksheets in each file, one for each visual field quadrant. The first column in each sheet is the eccentricity of each band. The second column is the mean pRF spread or local surface area of that eccentricity band. In the *-sigma.xls files the third and fourth columns are the length of the error bars, which correspond to the bootstrapped 95% confidence intervals. [file ncomms12110-s2.zip › Individuals/S10-area.png]

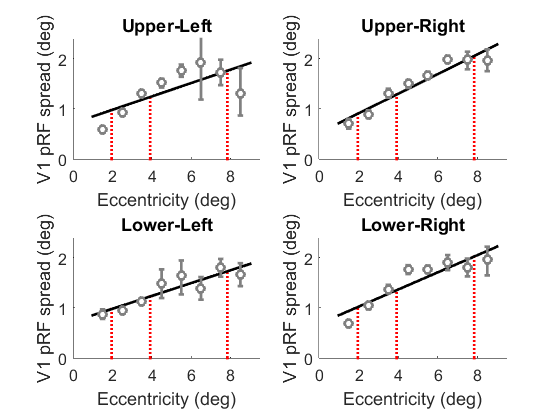

Supplement: Supplementary Data 1 — Each PNG file plots the mean V1 pRF spread (*-sigma.png) or the local surface area (*-area.png) in V1 for the four visual field quadrants against eccentricity bands that are 1° in width. The vertical dashed red lines indicate the eccentricities of the target stimuli in the psychophysical experiments. The solid black lines denote the fitted polynomial functions.The associated XLS files contain the mean pRF spread (*-sigma.xls) or local surface area (*-area.xls) in V1 plotted for each eccentricity band in these plots. There are four worksheets in each file, one for each visual field quadrant. The first column in each sheet is the eccentricity of each band. The second column is the mean pRF spread or local surface area of that eccentricity band. In the *-sigma.xls files the third and fourth columns are the length of the error bars, which correspond to the bootstrapped 95% confidence intervals. [file ncomms12110-s2.zip › Individuals/S10-sigma.png]

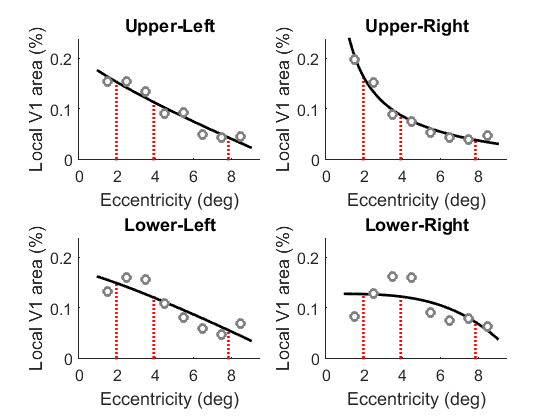

Supplement: Supplementary Data 1 — Each PNG file plots the mean V1 pRF spread (*-sigma.png) or the local surface area (*-area.png) in V1 for the four visual field quadrants against eccentricity bands that are 1° in width. The vertical dashed red lines indicate the eccentricities of the target stimuli in the psychophysical experiments. The solid black lines denote the fitted polynomial functions.The associated XLS files contain the mean pRF spread (*-sigma.xls) or local surface area (*-area.xls) in V1 plotted for each eccentricity band in these plots. There are four worksheets in each file, one for each visual field quadrant. The first column in each sheet is the eccentricity of each band. The second column is the mean pRF spread or local surface area of that eccentricity band. In the *-sigma.xls files the third and fourth columns are the length of the error bars, which correspond to the bootstrapped 95% confidence intervals. [file ncomms12110-s2.zip › Individuals/S2-area.png]

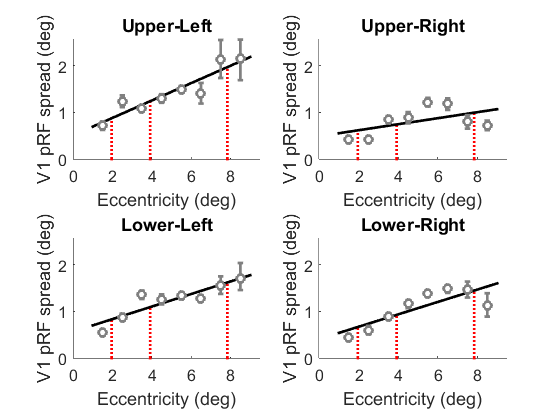

Supplement: Supplementary Data 1 — Each PNG file plots the mean V1 pRF spread (*-sigma.png) or the local surface area (*-area.png) in V1 for the four visual field quadrants against eccentricity bands that are 1° in width. The vertical dashed red lines indicate the eccentricities of the target stimuli in the psychophysical experiments. The solid black lines denote the fitted polynomial functions.The associated XLS files contain the mean pRF spread (*-sigma.xls) or local surface area (*-area.xls) in V1 plotted for each eccentricity band in these plots. There are four worksheets in each file, one for each visual field quadrant. The first column in each sheet is the eccentricity of each band. The second column is the mean pRF spread or local surface area of that eccentricity band. In the *-sigma.xls files the third and fourth columns are the length of the error bars, which correspond to the bootstrapped 95% confidence intervals. [file ncomms12110-s2.zip › Individuals/S2-sigma.png]

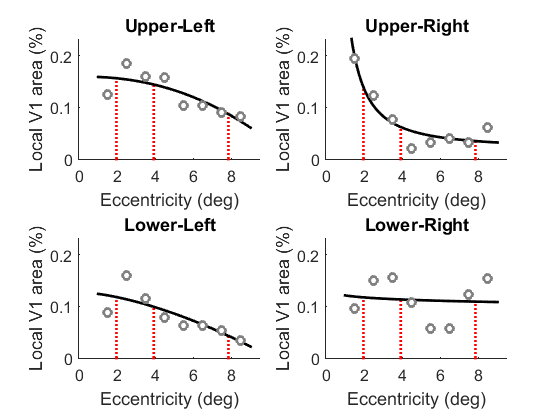

Supplement: Supplementary Data 1 — Each PNG file plots the mean V1 pRF spread (*-sigma.png) or the local surface area (*-area.png) in V1 for the four visual field quadrants against eccentricity bands that are 1° in width. The vertical dashed red lines indicate the eccentricities of the target stimuli in the psychophysical experiments. The solid black lines denote the fitted polynomial functions.The associated XLS files contain the mean pRF spread (*-sigma.xls) or local surface area (*-area.xls) in V1 plotted for each eccentricity band in these plots. There are four worksheets in each file, one for each visual field quadrant. The first column in each sheet is the eccentricity of each band. The second column is the mean pRF spread or local surface area of that eccentricity band. In the *-sigma.xls files the third and fourth columns are the length of the error bars, which correspond to the bootstrapped 95% confidence intervals. [file ncomms12110-s2.zip › Individuals/S3-area.png]

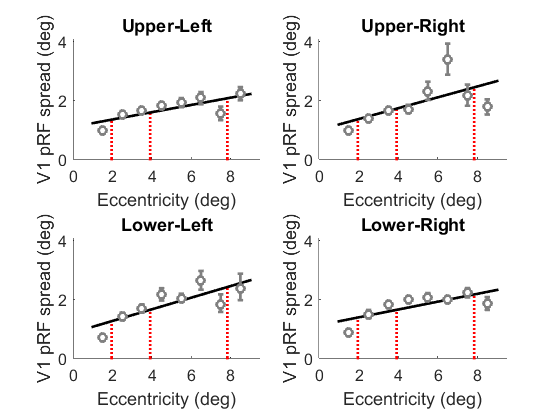

Supplement: Supplementary Data 1 — Each PNG file plots the mean V1 pRF spread (*-sigma.png) or the local surface area (*-area.png) in V1 for the four visual field quadrants against eccentricity bands that are 1° in width. The vertical dashed red lines indicate the eccentricities of the target stimuli in the psychophysical experiments. The solid black lines denote the fitted polynomial functions.The associated XLS files contain the mean pRF spread (*-sigma.xls) or local surface area (*-area.xls) in V1 plotted for each eccentricity band in these plots. There are four worksheets in each file, one for each visual field quadrant. The first column in each sheet is the eccentricity of each band. The second column is the mean pRF spread or local surface area of that eccentricity band. In the *-sigma.xls files the third and fourth columns are the length of the error bars, which correspond to the bootstrapped 95% confidence intervals. [file ncomms12110-s2.zip › Individuals/S3-sigma.png]

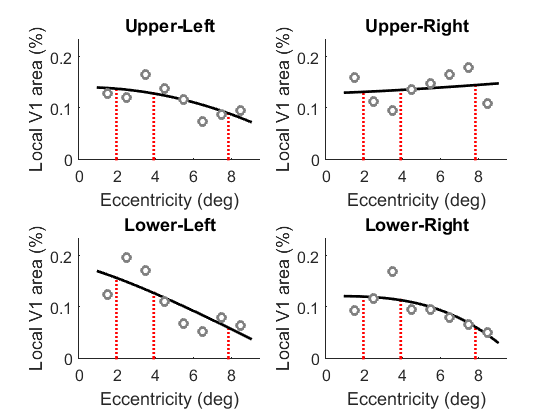

Supplement: Supplementary Data 1 — Each PNG file plots the mean V1 pRF spread (*-sigma.png) or the local surface area (*-area.png) in V1 for the four visual field quadrants against eccentricity bands that are 1° in width. The vertical dashed red lines indicate the eccentricities of the target stimuli in the psychophysical experiments. The solid black lines denote the fitted polynomial functions.The associated XLS files contain the mean pRF spread (*-sigma.xls) or local surface area (*-area.xls) in V1 plotted for each eccentricity band in these plots. There are four worksheets in each file, one for each visual field quadrant. The first column in each sheet is the eccentricity of each band. The second column is the mean pRF spread or local surface area of that eccentricity band. In the *-sigma.xls files the third and fourth columns are the length of the error bars, which correspond to the bootstrapped 95% confidence intervals. [file ncomms12110-s2.zip › Individuals/S4-area.png]

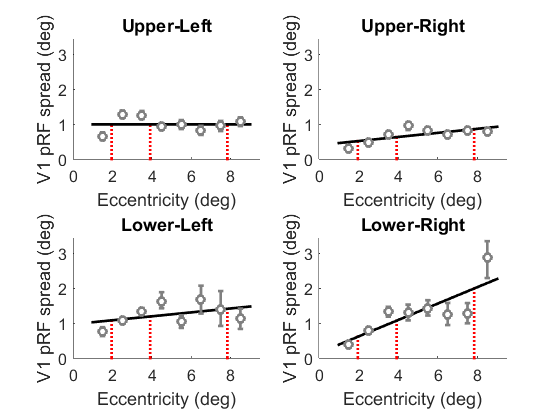

Supplement: Supplementary Data 1 — Each PNG file plots the mean V1 pRF spread (*-sigma.png) or the local surface area (*-area.png) in V1 for the four visual field quadrants against eccentricity bands that are 1° in width. The vertical dashed red lines indicate the eccentricities of the target stimuli in the psychophysical experiments. The solid black lines denote the fitted polynomial functions.The associated XLS files contain the mean pRF spread (*-sigma.xls) or local surface area (*-area.xls) in V1 plotted for each eccentricity band in these plots. There are four worksheets in each file, one for each visual field quadrant. The first column in each sheet is the eccentricity of each band. The second column is the mean pRF spread or local surface area of that eccentricity band. In the *-sigma.xls files the third and fourth columns are the length of the error bars, which correspond to the bootstrapped 95% confidence intervals. [file ncomms12110-s2.zip › Individuals/S4-sigma.png]

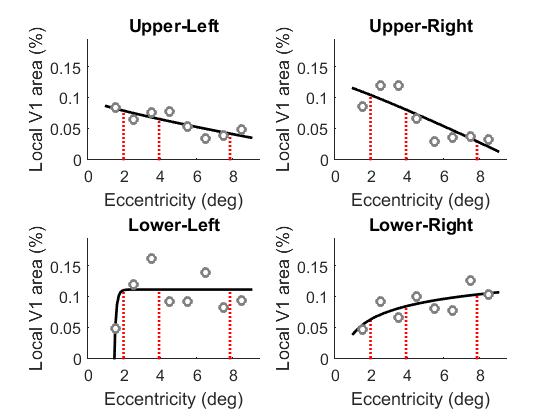

Supplement: Supplementary Data 1 — Each PNG file plots the mean V1 pRF spread (*-sigma.png) or the local surface area (*-area.png) in V1 for the four visual field quadrants against eccentricity bands that are 1° in width. The vertical dashed red lines indicate the eccentricities of the target stimuli in the psychophysical experiments. The solid black lines denote the fitted polynomial functions.The associated XLS files contain the mean pRF spread (*-sigma.xls) or local surface area (*-area.xls) in V1 plotted for each eccentricity band in these plots. There are four worksheets in each file, one for each visual field quadrant. The first column in each sheet is the eccentricity of each band. The second column is the mean pRF spread or local surface area of that eccentricity band. In the *-sigma.xls files the third and fourth columns are the length of the error bars, which correspond to the bootstrapped 95% confidence intervals. [file ncomms12110-s2.zip › Individuals/S5-area.png]

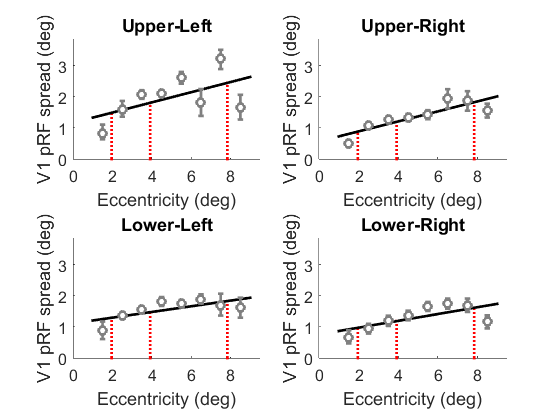

Supplement: Supplementary Data 1 — Each PNG file plots the mean V1 pRF spread (*-sigma.png) or the local surface area (*-area.png) in V1 for the four visual field quadrants against eccentricity bands that are 1° in width. The vertical dashed red lines indicate the eccentricities of the target stimuli in the psychophysical experiments. The solid black lines denote the fitted polynomial functions.The associated XLS files contain the mean pRF spread (*-sigma.xls) or local surface area (*-area.xls) in V1 plotted for each eccentricity band in these plots. There are four worksheets in each file, one for each visual field quadrant. The first column in each sheet is the eccentricity of each band. The second column is the mean pRF spread or local surface area of that eccentricity band. In the *-sigma.xls files the third and fourth columns are the length of the error bars, which correspond to the bootstrapped 95% confidence intervals. [file ncomms12110-s2.zip › Individuals/S5-sigma.png]

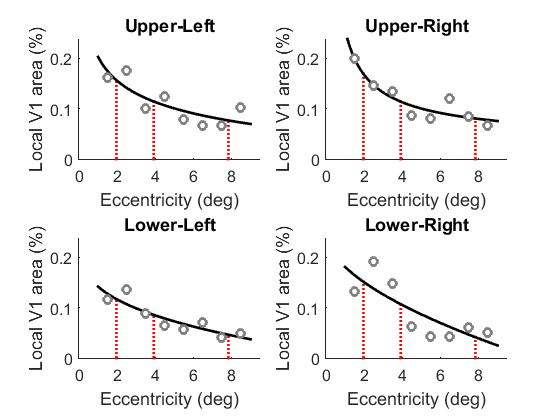

Supplement: Supplementary Data 1 — Each PNG file plots the mean V1 pRF spread (*-sigma.png) or the local surface area (*-area.png) in V1 for the four visual field quadrants against eccentricity bands that are 1° in width. The vertical dashed red lines indicate the eccentricities of the target stimuli in the psychophysical experiments. The solid black lines denote the fitted polynomial functions.The associated XLS files contain the mean pRF spread (*-sigma.xls) or local surface area (*-area.xls) in V1 plotted for each eccentricity band in these plots. There are four worksheets in each file, one for each visual field quadrant. The first column in each sheet is the eccentricity of each band. The second column is the mean pRF spread or local surface area of that eccentricity band. In the *-sigma.xls files the third and fourth columns are the length of the error bars, which correspond to the bootstrapped 95% confidence intervals. [file ncomms12110-s2.zip › Individuals/S6-area.png]

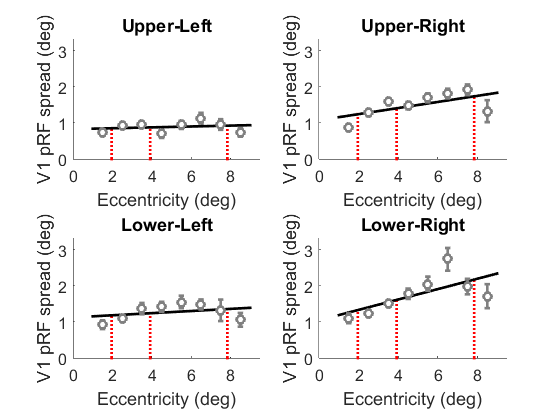

Supplement: Supplementary Data 1 — Each PNG file plots the mean V1 pRF spread (*-sigma.png) or the local surface area (*-area.png) in V1 for the four visual field quadrants against eccentricity bands that are 1° in width. The vertical dashed red lines indicate the eccentricities of the target stimuli in the psychophysical experiments. The solid black lines denote the fitted polynomial functions.The associated XLS files contain the mean pRF spread (*-sigma.xls) or local surface area (*-area.xls) in V1 plotted for each eccentricity band in these plots. There are four worksheets in each file, one for each visual field quadrant. The first column in each sheet is the eccentricity of each band. The second column is the mean pRF spread or local surface area of that eccentricity band. In the *-sigma.xls files the third and fourth columns are the length of the error bars, which correspond to the bootstrapped 95% confidence intervals. [file ncomms12110-s2.zip › Individuals/S6-sigma.png]

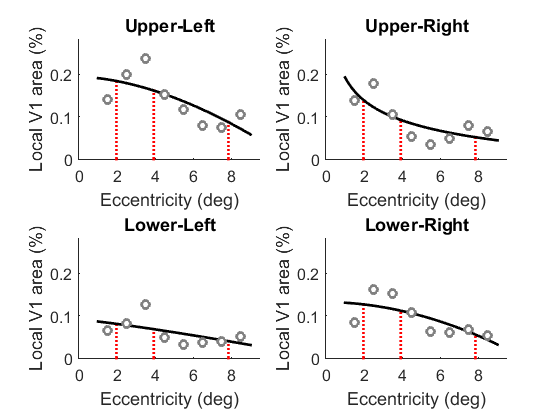

Supplement: Supplementary Data 1 — Each PNG file plots the mean V1 pRF spread (*-sigma.png) or the local surface area (*-area.png) in V1 for the four visual field quadrants against eccentricity bands that are 1° in width. The vertical dashed red lines indicate the eccentricities of the target stimuli in the psychophysical experiments. The solid black lines denote the fitted polynomial functions.The associated XLS files contain the mean pRF spread (*-sigma.xls) or local surface area (*-area.xls) in V1 plotted for each eccentricity band in these plots. There are four worksheets in each file, one for each visual field quadrant. The first column in each sheet is the eccentricity of each band. The second column is the mean pRF spread or local surface area of that eccentricity band. In the *-sigma.xls files the third and fourth columns are the length of the error bars, which correspond to the bootstrapped 95% confidence intervals. [file ncomms12110-s2.zip › Individuals/S7-area.png]

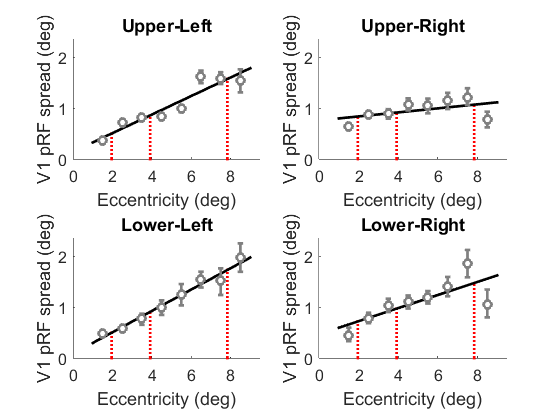

Supplement: Supplementary Data 1 — Each PNG file plots the mean V1 pRF spread (*-sigma.png) or the local surface area (*-area.png) in V1 for the four visual field quadrants against eccentricity bands that are 1° in width. The vertical dashed red lines indicate the eccentricities of the target stimuli in the psychophysical experiments. The solid black lines denote the fitted polynomial functions.The associated XLS files contain the mean pRF spread (*-sigma.xls) or local surface area (*-area.xls) in V1 plotted for each eccentricity band in these plots. There are four worksheets in each file, one for each visual field quadrant. The first column in each sheet is the eccentricity of each band. The second column is the mean pRF spread or local surface area of that eccentricity band. In the *-sigma.xls files the third and fourth columns are the length of the error bars, which correspond to the bootstrapped 95% confidence intervals. [file ncomms12110-s2.zip › Individuals/S7-sigma.png]

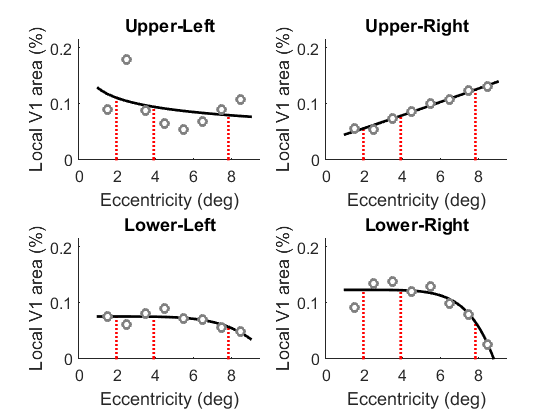

Supplement: Supplementary Data 1 — Each PNG file plots the mean V1 pRF spread (*-sigma.png) or the local surface area (*-area.png) in V1 for the four visual field quadrants against eccentricity bands that are 1° in width. The vertical dashed red lines indicate the eccentricities of the target stimuli in the psychophysical experiments. The solid black lines denote the fitted polynomial functions.The associated XLS files contain the mean pRF spread (*-sigma.xls) or local surface area (*-area.xls) in V1 plotted for each eccentricity band in these plots. There are four worksheets in each file, one for each visual field quadrant. The first column in each sheet is the eccentricity of each band. The second column is the mean pRF spread or local surface area of that eccentricity band. In the *-sigma.xls files the third and fourth columns are the length of the error bars, which correspond to the bootstrapped 95% confidence intervals. [file ncomms12110-s2.zip › Individuals/S8-area.png]

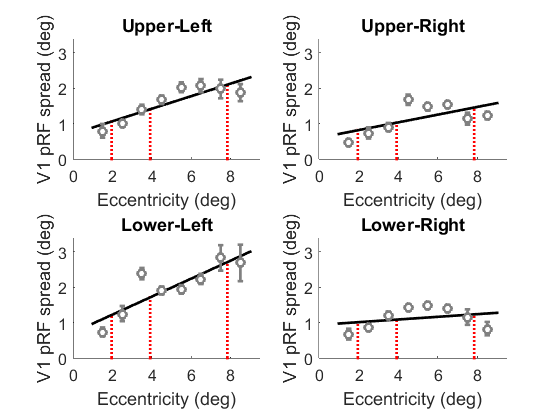

Supplement: Supplementary Data 1 — Each PNG file plots the mean V1 pRF spread (*-sigma.png) or the local surface area (*-area.png) in V1 for the four visual field quadrants against eccentricity bands that are 1° in width. The vertical dashed red lines indicate the eccentricities of the target stimuli in the psychophysical experiments. The solid black lines denote the fitted polynomial functions.The associated XLS files contain the mean pRF spread (*-sigma.xls) or local surface area (*-area.xls) in V1 plotted for each eccentricity band in these plots. There are four worksheets in each file, one for each visual field quadrant. The first column in each sheet is the eccentricity of each band. The second column is the mean pRF spread or local surface area of that eccentricity band. In the *-sigma.xls files the third and fourth columns are the length of the error bars, which correspond to the bootstrapped 95% confidence intervals. [file ncomms12110-s2.zip › Individuals/S8-sigma.png]

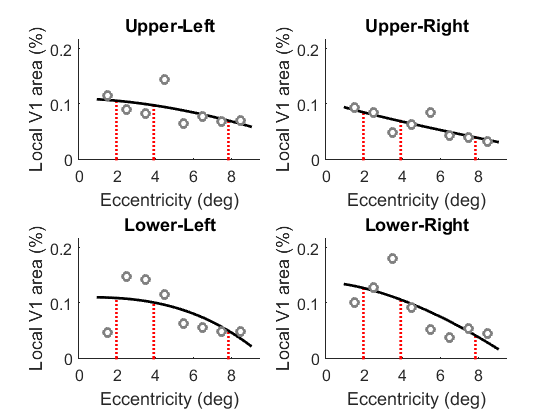

Supplement: Supplementary Data 1 — Each PNG file plots the mean V1 pRF spread (*-sigma.png) or the local surface area (*-area.png) in V1 for the four visual field quadrants against eccentricity bands that are 1° in width. The vertical dashed red lines indicate the eccentricities of the target stimuli in the psychophysical experiments. The solid black lines denote the fitted polynomial functions.The associated XLS files contain the mean pRF spread (*-sigma.xls) or local surface area (*-area.xls) in V1 plotted for each eccentricity band in these plots. There are four worksheets in each file, one for each visual field quadrant. The first column in each sheet is the eccentricity of each band. The second column is the mean pRF spread or local surface area of that eccentricity band. In the *-sigma.xls files the third and fourth columns are the length of the error bars, which correspond to the bootstrapped 95% confidence intervals. [file ncomms12110-s2.zip › Individuals/S9-area.png]

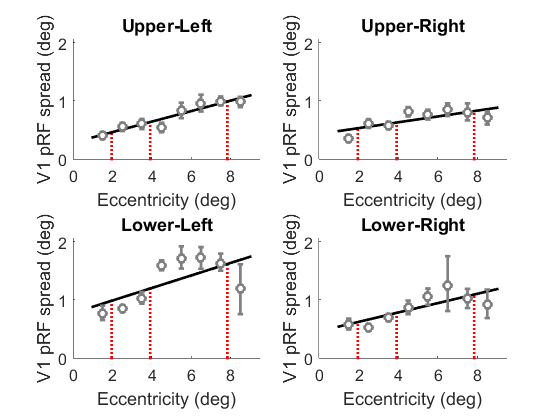

Supplement: Supplementary Data 1 — Each PNG file plots the mean V1 pRF spread (*-sigma.png) or the local surface area (*-area.png) in V1 for the four visual field quadrants against eccentricity bands that are 1° in width. The vertical dashed red lines indicate the eccentricities of the target stimuli in the psychophysical experiments. The solid black lines denote the fitted polynomial functions.The associated XLS files contain the mean pRF spread (*-sigma.xls) or local surface area (*-area.xls) in V1 plotted for each eccentricity band in these plots. There are four worksheets in each file, one for each visual field quadrant. The first column in each sheet is the eccentricity of each band. The second column is the mean pRF spread or local surface area of that eccentricity band. In the *-sigma.xls files the third and fourth columns are the length of the error bars, which correspond to the bootstrapped 95% confidence intervals. [file ncomms12110-s2.zip › Individuals/S9-sigma.png]
